# Complete and Draft Genome Sequences of Amino Acid-Producing Corynebacterium glutamicum Strains ATCC 21799 and ATCC 31831 and Their Genomic Islands

**DOI:** 10.1128/MRA.00430-20

**Published:** 2020-08-06

**Authors:** Hideo Kawaguchi, Takashi Sazuka, Akihiko Kondo

**Affiliations:** a Graduate School of Science, Technology, and Innovation, Kobe University, Kobe, Japan; b Bioscience and Biotechnology Center, Nagoya University, Nagoya, Japan; c Engineering Biology Research Center, Kobe University, Kobe, Japan; Loyola University Chicago

## Abstract

We determined the complete and draft genome sequences of two strains of Corynebacterium glutamicum and revealed their genomic islands (GEIs). The two strains, ATCC 21799 and ATCC 31831, were found to have 3,079 and 3,109 coding sequences, respectively, with 13 GEIs each not present in the reference strain, ATCC 13032.

## ANNOUNCEMENT

Corynebacterium glutamicum is a Gram-positive soil microorganism (1) widely used for industrial amino acid production (2, 3). Although the genomes of several C. glutamicum strains have been elucidated (4–7), their genomic islands (GEIs) have not been comprehensively reported. Therefore, we determined the complete and draft genome sequences of two C. glutamicum strains, ATCC 21799 and ATCC 31831, and predicted GEIs not present in the reference strain, ATCC 13032.

Two C. glutamicum strains purchased from ATCC (Manassas, VA) were cultivated aerobically in brain heart infusion broth (Merck, Darmstadt, Germany) at 30°C. After 16 h of cultivation, the genomic DNA was extracted from these strains using a Nucleobond AXG system (TaKaRa Bio, Shiga, Japan). DNA sequencing was performed using the PacBio RS II system (Pacific Biosciences, Menlo Park, CA) with DNA sequencing reagent 4.0 v2. A single SMRTbell library was prepared according to the manufacturer’s instructions and was sheared to 15 kb using BluePippin size selection system v10 (Sage Science, Beverly, MA). The genomes were assembled with Hierarchical Genome Assembly Process v2.3.0 (8), and filtering was based on a threshold of 0.80 for minimum polymerase read quality. GEIs were predicted using GIPSy v1.1.2 software (9), and maps of the circular genomes of C. glutamicum showing GEIs were generated using BLAST Ring Image Generator (BRIG) v0.95 software (10) for the reference strain, ATCC 13032 (GenBank accession number BA000036.3). In all analyses, default parameters were used except when otherwise noted.

For strain ATCC 21799, 107,747 filtered reads with an *N*_50_ value of 14,464 bp were assembled into one contig, yielding a 3,332,273-bp complete sequence. However, for strain ATCC 31831, 144,465 filtered reads with an *N*_50_ value of 18,854 bp were assembled into two contigs, namely, contigs 1 and 2 (3,302,680 bp and 29,004 bp, respectively), yielding a 3,311,684-bp draft genome sequence. Coverage depths were 282× and 301× with average G+C contents of 54.3% and 54.0% for strains ATCC 21799 and ATCC 31831, respectively. Genome sequences were automatically annotated using the genome annotation pipeline DFAST (11), yielding 3,079 and 3,109 coding sequences (CDSs), 65 and 62 tRNAs, and 18 and 21 rRNAs, respectively, for strains ATCC 21799 and ATCC 31831.

**FIG 1:**
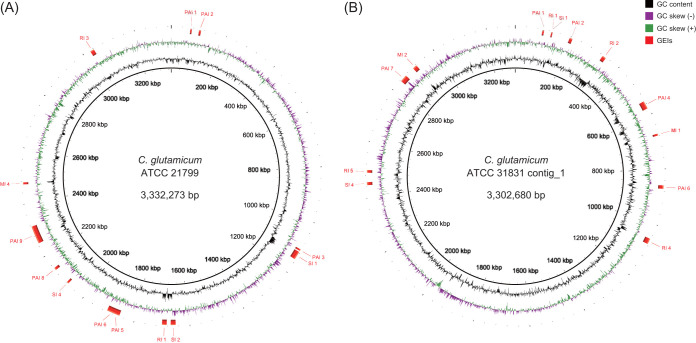
Maps of the circular genomes of Corynebacterium glutamicum ATCC 21799 (A) and ATCC 31831 (B) showing genomic islands (GEIs) predicted for the C. glutamicum type strain ATCC 13032 and regions of genome plasticity. The figure was generated with C. glutamicum ATCC 13032 as the reference strain using the software BLAST Ring Image Generator (BRIG) (10). GEIs are classified as putative pathogenicity islands (PAI), resistance islands (RI), metabolic islands (MI), and symbiotic islands (SI).

The genomes of strains ATCC 21799 and ATCC 31831 had 13 GEIs each. In strain ATCC 21799, pathogenicity island 9 (PAI 9) was the largest GEI (65 kb) and had transposases at both ends (Fig. 1A). This region consisted of 55 coding DNA sequences (CDSs) (KaCgl20770 to KaCgl21310), and 80% of them showed a deviation in codon usage from the standard value of 0.95. In strain ATCC 31831, metabolic island 2 (MI 2) (KbCgl27180 to KbCgl27280) is involved in an exclusive gene cluster responsible for l-arabinose utilization (12) with distinct codon usage patterns (Fig. 1B). In conclusion, the two GEIs of C. glutamicum are unique and structurally discrete sequences that most likely arose independently during evolution by horizontal gene transfer.

### Data availability.

The complete genome sequence of strain ATCC 21799 and the draft genome sequences of strain ATCC 31831 have been deposited under DDBJ/ENA/GenBank accession number AP022856.1 and numbers BLRJ01000001.1 and BLRJ01000002.1, respectively. The raw reads were deposited under SRA accession numbers DRR232384 and DRR232383, respectively.
